# Connecting High School Students With Nature – How Different Guided Tours in the **Zoo** Influence the Success of Extracurricular Educational Programs

**DOI:** 10.3389/fpsyg.2020.01804

**Published:** 2020-08-04

**Authors:** Matthias Winfried Kleespies, Jennifer Gübert, Alexander Popp, Nicola Hartmann, Christian Dietz, Tanja Spengler, Martin Becker, Paul Wilhelm Dierkes

**Affiliations:** ^1^Department of Biology, Bioscience Education and Zoo Biology, Goethe University Frankfurt, Frankfurt, Germany; ^2^Department of Chemistry, Didactics of Chemistry, Goethe University Frankfurt, Frankfurt, Germany; ^3^Opel-Zoo, Kronberg, Germany

**Keywords:** connection to nature, zoo education, environmental education, INS, interest in animals

## Abstract

Zoos attract millions of visitors every year, many of whom are schoolchildren. For this reason, zoos are important institutions for the environmental education of future generations. Empirical studies on the educational impact of environmental education programs in zoos are still rare. To address this issue, we conducted two studies: In study 1, we investigated students’ interests in different biological topics, including zoos (*n* = 1,587). Data analysis of individual topics revealed large differences of interest, with advanced students showing less interest in zoos. In study 2, we invited school classes of this age group to visit different guided tours at the zoo and tested connection to nature before and after each educational intervention (*n* = 608). The results showed that the guided tours are an effective tool to raise students’ connection to nature. Add-on components have the potential to further promote connection to nature. The education programs are most effective with students with a low initial nature connection.

## Introduction

In our modern society, zoos and aquariums play an emerging role as educational and nature conservation centers with extensive educational programs. This important role becomes obvious when looking at the number of zoo visitors. The European Association of Zoos and Aquaria reported 140 million visitors each year (Griffith, 2017), and the VdZ, the oldest zoo association in the world, with currently 71 members, welcomes more than 43 million visitors each year (Kögler et al., 2020). At a global level, it can be assumed that more than 700 million people visit zoos and aquariums yearly (Gusset and Dick, 2011). The main objectives of zoos are often summarized as research, conservation, education, and entertainment (Churchman, 1984). In recent times, zoos focus more on conservation and education and have become more and more “Centers of Conservation and Caring” (Rabb, 2004). Patrick et al. (2007) analyzed the mission statements of 137 zoos in the United States and discovered that 97% of the zoos had education as their main theme in their mission statement. On the second place was conservation, which was mentioned in 85% of the zoo statements. Both zoos and visitors believe that zoos should be a place for visitors and in particular for schoolchildren to learn (Roe et al., 2014). To accomplish this goal, the majority of zoos and aquariums offer education programs for their visitors, for example, guided tours, keeper talks, and information materials on the spot and on the internet, as well as special education courses for school groups. Particularly in the field of environmental education, school classes represent an important target group (Andersen, 2003). However, zoos should demonstrate that a visit to the zoo contributes to an understanding of biodiversity and nature conservation issues and ideally leads to proenvironmental behavior.

### Evaluating the Educational Impact in Zoos

The main goal of most environmental education programs is to change participants’ behavior to a more sustainable and environmentally friendly behavior. To achieve this goal and to evaluate the success of environmental education programs, there are different approaches in environmental education research. One is the focus on environmental attitudes, which are defined as concern for the environment or environmental issues (Gifford and Sussman, 2012) and can be actively promoted through environmental education programs (Johnson and Manoli, 2010; Liefländer and Bogner, 2014; Schmitz and Da Rocha, 2018). The connection between environmental attitudes and environmentally friendly behavior can be explained, for example, by the value–belief–norm theory. In this theory, attitudes or beliefs are regarded as influencing factors on personal norms, which in turn directly influence behavior (Stern, 2000). This makes environmental attitudes a strong predictor of ecological behavior (Kaiser et al., 1999).

In addition to environmental attitudes, knowledge about the environment also plays an important role in environmental education. Knowledge is often associated with proenvironmental behavior (Hines et al., 1987; Kaiser and Fuhrer, 2003; Frick et al., 2004; Latif et al., 2013; Vicente-Molina et al., 2013; Zsóka et al., 2013). However, this old paradigm is criticized by some authors (Otto and Pensini, 2017). Ogden and Heimlich (2009) call it a myth that knowledge turns into a change of behavior, and Moss et al. (2017) have recently analyzed the connection between knowledge and proenvironmental behaviors with a large sample size. While they proved a positive effect of knowledge on proconservation behavior, this effect was small.

Although the evaluation of environmental education programs is important to further develop and improve programs, to increase understanding, and to draw conclusions for new programs (Patton, 2008), there is not yet sufficient research in this field. For example, from 56 examined conservation education reports, only 45.6% evaluated their programs in some form, and even fewer (23.1%) used summative evaluation (Norris and Jacobson, 1998).

When zoos evaluate environmental education programs, they usually focus on two core areas of environmental education described above: on the one hand, visitors learn and gain in knowledge and, on the other hand, visitors’ change in attitudes. Wagner et al. (2009), for example, surveyed more than 700 zoo members and visitors with a pre–post instrument. The measurement showed a great gain in conservation–knowledge and conservation–motivation by a zoo visit, leading to the conclusion that zoos are able to positively influence knowledge, attitudes, skills, and behavior. A similar positive effect on visitors’ knowledge, attitudes, and behavior was observed for dolphin shows at six different zoos (Miller et al., 2013). Additionally, to the short-term effects, Miller et al. (2013) verified the long-term outcomes after 3 months. Visitors showed a short-term increase in all mentioned categories. The follow-up test revealed that knowledge is preserved, and more environmentally friendly behavior is applied. An even longer-lasting effect was proven by Falk et al. (2007) showing that a year after the zoo visit the majority of the visitors still had a positive change of knowledge. Another interesting approach to observe the change in behavior after a zoo visit was used by MacDonald (2015). As a part of an animal presentation in the Wellington zoo, she told zoo visitors that keeping cats indoor at night helps to protect the native wildlife. Six weeks after the zoo trip, 57% of the participants reported to have implemented the proenvironmental behavior, and the number even increases to 100% when the visitors were asked to sign a pledge card that was displayed at the zoo.

For guided tours and unguided zoo visits, the results in conservation learning are not as clear. Jensen (2014) observed a group of 7–15-years-old students. While 41% of the pupils showed a positive effect when learning about nature conservation on a guided tour, the effect on an unguided tour was slightly lower, and approximately only one-third showed a positive effect. For a detailed literature review of conservation education in zoos, we recommend Nygren and Ojalammi (2018).

**FIGURE 1:**
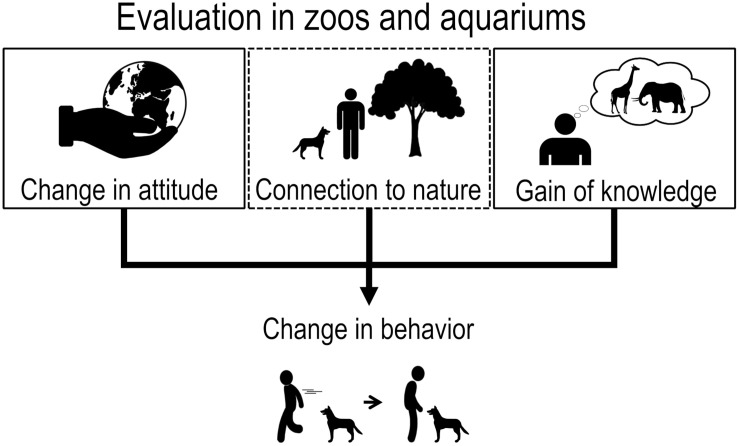
In addition to changes in attitude and knowledge, connection to nature represents a further, major factor in achieving a change in behavior.

From the perspective of zoos, the focus on knowledge gain and attitude change makes sense, as these factors have a strong influence on environmental behavior, and their promotion contributes to achieving the main goal: behavioral change to proenvironmental behavior. Nevertheless, current zoo evaluation usually lacks another important concept that has great potential and is well known in environmental education research: connection to nature (Figure 1).

### Connection to Nature and Zoos

Although the connection to nature has been a topic of environmental research for years, there is no clear and generally accepted definition of the concept. While some definitions of connection to nature emphasize the emotional component of the construct (e.g., Mayer and Frantz, 2004; Nisbet et al., 2009), others focus on the relationship between nature and the personal identity (Clayton, 2003). In his description of the concept of inclusion of nature, Schultz (2002) describes three dimensions. The cognitive component includes the connection to nature, the affective component deals with the question whether people care about nature, and the behavioral component explains the motivation to protect nature and to behave in an environmentally friendly way. In our research, we focus in particular on the cognitive component of the construct, the connection to nature. Schultz defines connection to nature as “the extent to which an individual includes nature within his/her cognitive representation of self” (Schultz, 2002). It is often used to evaluate the success of programs in environmental education. In recent years, different scales were created to measure connection to nature. For example, the Connectedness to Nature Scale by Mayer and Frantz (2004), the Nature Relatedness Scale by Nisbet et al. (2009), the Environmental Identity Scale by Clayton (2003), and the Implicit Association Test by Schultz et al. (2004).

Connection to nature is important for various reasons: The link between connection to nature and proenvironmental behavior has been proven in a large number of research papers. People with higher connection to nature report more environmentally friendly behavior and attitudes and were more concerned about ecological problems (Mayer and Frantz, 2004; Nisbet et al., 2009; Frantz and Mayer, 2014; Geng et al., 2015). Thus, the strengthening of a person’s bond to nature leads to an increase of conservation performance (Kaiser et al., 2008). Especially for children, connection to nature influences the willingness to spend time in nature (Cheng and Monroe, 2012). On the other hand, a lack of proenvironmental behavior leads to a decrease of connection to nature (Frantz et al., 2005). A strengthened connection to nature has also an effect on well-being; living in a place with more green spaces decreases the mental distress and increases the personal well-being (Nisbet et al., 2010; White et al., 2013). In addition, the psychological well-being and social well-being correlate positive with connection to nature (Howell et al., 2011; Piccininni et al., 2018). Hence, connection to nature is a significant predictor of well-being (Loureiro and Veloso, 2014). An analogous relationship to well-being is also described for vitality. Spending time in nature is associated with an increase in vitality (Ryan et al., 2010; Cervinka et al., 2012; Capaldi et al., 2014), and thus, the connection to nature contributes to psychological health (Nisbet et al., 2010).

In summary, these examples show that connection to nature is an important factor that needs to be promoted through environmental education programs. From the zoos’ point of view, too, strengthening the connection to nature should be an important goal. Increasing the visitors’ connection to nature helps to achieve the primary goal of zoos, namely, the promotion of proenvironmental behavior.

Research on the topic of connection to nature is underrepresented in the zoo context, and the few reported results are inconsistent. On the one hand, studies reveal a positive effect of a zoo visit on the connection to nature (Falk et al., 2007; Schultz and Tabanico, 2007; Clayton et al., 2014). On the other hand, this effect is not confirmed by Bruni et al. (2008), or even a small decrease is reported by Sattler (2016). At present, there are still many open research questions regarding connection to nature and zoos.

In this article, we have proceeded in two steps. Study 1 forms the basis for study 2, which aimed to identify the age group of students with the least interest in animals and zoos. For this purpose, more than 1,600 students of different grades were surveyed in a cross-sectional study. For study 2, the age group with the least interest in zoos or zoo animals from study 1 was selected to experience guided zoo tours with these students. The aim was to answer the question whether the most basic environmental education program at a zoo, a guided tour, has the potential to affect participants’ connection to nature. Additionally, we wanted to test whether it is possible to increase the outcome by small add-ons to the tour.

## Study 1

To explain the interest of a person, there are different approaches in psychology. Special attention is given to the person–object theory, which was especially influenced by Schiefele (1991, 1992), Prenzel (1992), and Krapp (1993, 1999). According to this theory, the environment consists of subareas that can be more or less separated from each other. These are called objects. They include, for example, living beings, events, connections, conditions, and so on. When a person deals with such an object, he/she establishes a relationship that varies in quality and intensity. An outstanding person–object relationship is called interest (Krapp, 1992a). Interest is essential for academic success and plays a crucial role in the learning process. Because interest on certain topics declines over time, it is necessary to promote it (Harackiewicz et al., 2016). A positive correlation between grades and interest shows the effect of interest on students’ performance at school (Krapp, 1992b). Instead of only reproducing knowledge, interest leads to independent participation (Schiefele, 1991). Schreiner and Sjøberg (2004) initialized the ROSE study in order to observe students’ attitude, views, and interest toward science and technology. As part of this large-scale project and cooperation between different researchers, students in more than 40 countries were surveyed with a questionnaire containing almost 250 questions on science and technology (Sjøberg and Schreiner, 2010). In three sections with a total of more than 100 items, students should rate on a four-point Likert scale how interested they are in learning about different aspects of science and technology. An important objective of this study was to provide empirical evidence and discussion students’ interest to improve the quality, relevance, and attentiveness for science and technology (Schreiner and Sjøberg, 2004). The ROSE study discovered an overall positive attitude to science and technology. While boys tend to be more interested and positive over all, girls showed mainly interest on environmental issues (Sjøberg and Schreiner, 2010).

Because interest is an important factor, we want to determine the age group of students with the least interest in animals and zoo topics in study 1.

### Materials and Methods

#### Measurement

Similar to the interest items used in the ROSE study (Schreiner and Sjøberg, 2004), we tested 15 biology-related interest items that had to be evaluated by students on a four-point Likert scale (Table 1). While the ROSE study focused on the interest in science and technology in general, our items focus on biological topics (such as plants, animals, and environment).

**TABLE 1:** Results of the PCA with varimax rotation.

		**1**	**2**	**3**	**4**
A1	What zoos do for protection of species	0.807			
A2	Animals in other areas of the world	0.789			
A3	Animals in the zoo	0.784			
A4	Behaviors of animals	0.712			
A5	How to protect endangered species	0.613			0.319
P1	Plants in my environment		0.747		
P2	How plants grow and multiply		0.738		
P3	Plants and their benefits for humans		0.694		
P4	How plants produce food		0.647		
H1	What you have to eat to stay healthy			0.865	
H2	How to train to keep the body fit and healthy			0.845	
H3	Nutrition of humans			0.671	
E1	The greenhouse effect and how humans can change the situation				0.819
E2	How to preserve and protect biodiversity in the world	0.362			0.631
E3	Habitat earth				0.622

*Only factor loadings > 0.3 were listed.*

#### Participants

Data collection took place from September 2013 to July 2015. Overall, 1,587 students of different grades participated in our survey. Four hundred eleven were fifth graders, 62 sixth graders, 418 seventh graders, and 448 nine graders. In 10th or 11th grade, 41 students attended; in 12th grade, 174; and in 13th grade, 33 students. The participation in the study was voluntary and anonymous. For underage participants, the parents had to sign a letter of agreement, and private policy has been respected.

#### Analysis

All statistical analysis was executed using IBM SPSS 24. To investigate the relationship between the 15 interest items, a principal component analysis (PCA) was performed, and to prove sampling adequacy, the Bartlett test and the Kaiser–Meyer–Olkin (KMO) test were applied. For the separated factors, Cronbach α was calculated to determine reliability and internal consistency.

### Results

The KMO test approved the sampling adequacy (KMO = 0.873), and the Bartlett test was highly significant (*p* < 0.001), fulfilling the conditions for a factor analysis.

**FIGURE 2:**
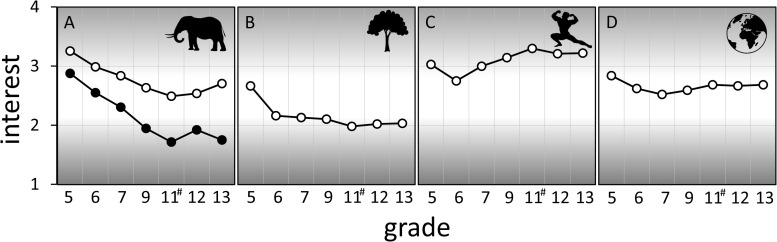
Development of the four factors of the PCA analysis in the school career. **(A)** Factor 1 (animals) was divided into two graphs according to the content statement. The upper graph contains the items concerning animals in general (A2; A4–A6, closed circles); the lower graph, the items concerning animals and zoo (A1 and A3, open circles). **(B)** Factor 2 (plants, P1–P4), **(C)** Factor 3 (health, H1–H3), **(D)** Factor 4 (environment, E1–E3). Scale from 1 = no interest to 4 = high interest. ^#^Students in the 10th and 11th were combined because of the small sample size.

The PCA with oblique rotation showed a four-factor solution for the 15 items (Table 1). Factor 1 (animals, A1–5) accounted for 31.64% of the variance and had an α = 0.833. The second factor (plants, P1–4) explained 13.59% of the variance with an α = 0.751. Factor 3 (health, H1–3) accounted for additional 8.84% (α = 0.732), and factor 4 (environment, E1–3) for 7.49% (α = 0.635) of the variance. For the four factors found, the changes in the dependency of the age groups recorded were different. The interest in animals shows a significant decrease after the sixth grade compared to the initial interest in the fifth grade. A similar result could also be observed for the interest in plants. Interest declines significantly after the fifth grade and remains at a similarly low level for the rest of the school career. In contrast, the health factor and the environmental factor show consistently high levels. Although there are also significant short-term deviations here, the interest at the end of school career does not differ from the initial interest in the fifth grade (Figure 2 and Table 2).

**TABLE 2:** Average scores of the different factors, divided by grades.

	**5**	**6**	**7**	**9**	**11^#^**	**12**	**13**
Animals (general)	3.26	2.99	2.84***	2.64***	2.49***	2.54***	2.71**
Animals (zoo)	2.88	2.55	2.31***	1.95***	1.72***	1.92***	1.75***
Plants	2.67	2.16***	2.13***	2.11***	1.98***	2.02***	2.04***
Health	3.03	2.75*	3.00	3.15	3.30	3.21*	3.22
Environment	2.84	2.62	2.52***	2.59***	2.69	2.67	2.69

Significant changes to the initial interest of the fifth grade are marked with an asterisk (*p < 0.05, **p < 0.01, ***p < 0.001). A detailed breakdown of the individual items by grades can be found in Table A1. The pairwise comparison between the fifth class (as initial interest) and the higher classes was carried out using a post hoc test. To avoid an inflated α value, a Bonferroni correction (with 6) was performed. ^#^Students in the 10th and 11th grades were combined because of the small sample size.

### Discussion

The results reveal a topic-related, different development of biological interests during the course of the school career. Previous studies showed that students even from different countries and cultures have the highest interest in biological questions dealing with human health, reproduction, or genetics (Hagay et al., 2013). Similar results are obtained in this study, showing that students start with a high and lasting interest on questions about human health in fifth grade (Figure 2C). Environmental protection is becoming an increasingly important topic in our society, and the increasing number of environmental education programs offered is an indicator of this (Thomas, 2014). The high level of interest, particularly among young adults, is also reflected in the high level of participation in current environmental protection movements such as “Fridays for Future.” In this context, an emotional approach to nature conservation also appears to be an important predictor of proenvironmental behavior (Kals et al., 1999). Our environmental component has a comparatively high value compared to the other groups, which shows only a slight decline in the school career during puberty (Figure 2D). This “dip” in interest has been observed before and can be explained by the brain and cognitive development of the students in this time period (Olsson and Gericke, 2016). During adolescence, changes in the brain occur, which can lead to cognitive and behavioral changes. These changes are often explained by the restructuring of the prefrontal cortex and the increasing communication of the prefrontal cortex with other brain regions (Steinberg, 2005).

The overall lower interest in plants, as well as the strong decrease in puberty (Figure 2B), is also described in earlier studies (Wandersee, 1986; Strgar, 2007). The term “plant blindness” was also coined in this context and expresses the fact that plants are often overlooked, and people have less knowledge about plants (Wandersee and Schussler, 1999). In comparison, animals show a higher interest, especially among younger pupils. The loss of interest in this regard depends on the thematic focus. Topics with a zoo focus experience a higher loss of interest during the school career than other zoological topics (Figure 2A). In addition to the loss of interest in various topics with increasing age, several studies have also shown that older students are less connected to nature than younger students (e.g., Schultz and Tabanico, 2007; Sattler, 2016; Braun and Dierkes, 2017). Based on these results, the targeted development, implementation, and evaluation of environmental education programs for high school students are an important task (study 2).

## Study 2

Study 1 showed that advanced students have a lower interest in animals and zoos. While interest in a topic can lead to a person becoming more engaged, more motivated, and showing better learning outcomes (Harackiewicz et al., 2016), disinterest leads to an opposite effect. For this reason, various zoo tours were conducted for high school students within the framework of study 2. In order to evaluate the success of the guided tours of the zoo with these groups of students, the connection to nature was measured and analyzed. The study deals in particular with the detailed comparison of regular tours and tours that included a special extension. For this purpose, the spectrum of zoo pedagogical possibilities was largely taken into account. Thus, there were guided tours with subsequent feeding, conversations with keepers, or a look behind the scenes. The aim was to find out which small add-on has the potential to further promote connection to nature in comparison to the standard tour and thus to develop tips to help zoos and organizers of environmental education programs to promote connection to nature in a targeted manner.

### Materials and Methods

**FIGURE 3:**
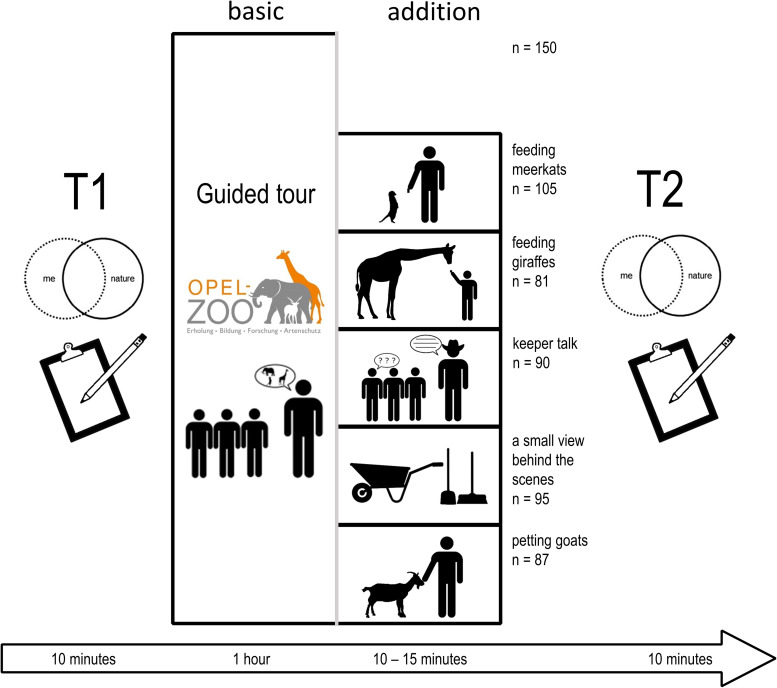
Study design. The environmental education program at the zoo included a guided tour, which could be supplemented by various add-ons. Before and after the tour, the participants filled out a questionnaire in which the connection to nature was determined.

The guided tours took place at the Opel Zoo in Kronberg (Germany). The main emphasis of the tour was to show the biodiversity of African mammals. The tour included stops at the Rothschild’s giraffe (*Giraffa camelopardalis rothschildi*), dwarf mongoose (*Helogale parvula*), meerkat (*Suricata suricatta*), Indian porcupine (*Hystrix indica*), common warthog (*Phacochoerus africanus*), barbary macaque (*Macaca sylvanus*), and African elephant (*Loxodonta africana*) enclosures. The guided tour focuses on biological and ecological facts, the adaptation of the animals to their habitat, social behavior, and threat category. Before and after the tour, connection to nature was measured to detect possible changes. The use of a personal code allowed comparison before and after the intervention. The regular tours were supplemented with short-term add-ons (10–15 min), and the respective effects on connection to nature were measured. The add-ons included giraffe feeding, meerkat feeding, a keeper talk, a look behind the scenes, or petting goats in the children’s zoo. The groups did not know about the additions before, and each group attended only one add-on (Figure 3).

**Feeding Giraffes:** Depending on the weather, the feeding of the giraffes took place in the hall of the giraffe enclosure or outside on the savanna facility. The students were allowed to touch and to feed the animals from their hand with crisp bread.

**Feeding Meerkats:** The feeding of the meerkats took place at the meerkat enclosure with mealworms. In order to avoid injuries from meerkat bites, the students were not allowed to touch or feed them out of their hands.

**Keeper Talk:** During the keeper talk, the students had the opportunity to ask their questions to a zookeeper and hear some firsthand experiences about the work with the animals in a zoo.

**Small View Behind the Scenes:** The view behind the scenes included a part of the elephant house that is inaccessible for the public view, including the elephant food and tools for cleaning the enclosure.

**Petting Zoo:** The students had the opportunity to go in the public petting zoo to pet different animals. Most of the animals in the petting zoo are West African pygmy goats, but there are also some sheep and a donkey.

#### Measurement of Connection to Nature

We used the Inclusion of Nature in Self-scale (INS) by Schultz (2002) to measure connection to nature. The INS is a graphical single item question, which consists of seven different pairs of circles, with one circle representing the person himself or herself and the other representing nature. The circle pairs differ in the amount of overlap representing different stages of connection to nature (Figure A1). The participants had to choose the circle pair that best describes their relationship to nature. Every questionnaire had an individual code, which made it possible to compare T1 and T2 of each student.

#### Participants

The groups in our survey registered for the guided tours by e-mail. The offer was aimed only at high school students in the 12th and 13th grades. Our sample consisted of 608 students, and participation on our survey was voluntary. For participants under the legal age, parents had to sign a letter of agreement, and privacy policy has been respected. One hundred fifty students participated in a guided tour without an addition, 105 attended in a guided tour with feeding meerkats, and 81 in a tour with feeding giraffes. Ninety students got a guided tour with a keeper talk, 95 with a view behind the scenes, and 87 attended in a tour with petting goats. Data were collected between December 2018 and May 2019.

#### Analysis

For the statistical analysis, we used IBM SPSS 24. To simplify the analysis and to get more meaningful results, we divided the connection to nature scores in three groups, depending on the initial INS score; INS scores from 1 to 3 were classified as low connection to nature, INS scores of 4 as medium connection to nature, and scores from 5 to 7 as high connection to nature. To examine the difference between T1 and T2, the Wilcoxon signed ranks test was used, after the Kolmogorov–Smirnov test was highly significant for all test groups on both measurements (*p* < 0.001). When the Wilcoxon test showed significant results between T1 and T2, the effect size (*r*) according to Fritz et al. (2012) was calculated, using the formula *r* = $\frac{z}{\sqrt{N}}$ for non-parametric data. For a comparability with other studies, we converted *r* in Cohen *d* using the formula *d* = $\frac{2\,r}{\sqrt{1-r^{2}}}$ by Borenstein et al. (2009).

### Results

**FIGURE 4:**
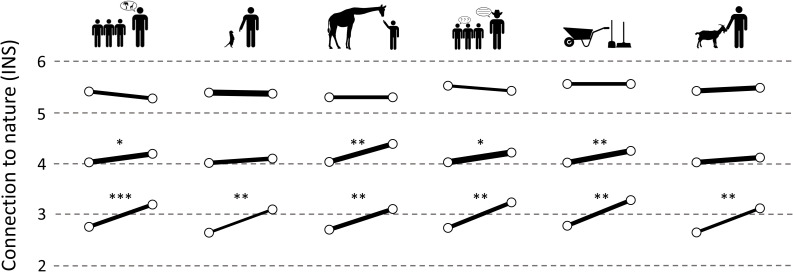
Immediate shifts in reported INS scores within the six different experimental groups. The horizontal lines reflect the levels of connection to nature (INS scores). The stroke width of the graphs is proportional to the number of students being represented in the subgroups. Only significant shifts are marked: *p < 0.05, **p < 0.01, ***p < 0.001.

The groups with high initial INS scores did not show a significant change in any of the six test setups. The groups with low connection exhibited a highly significant increase after the tour, which showed similar effect sizes for almost all groups (∼*d* = 1.4). An exception was found within the petting zoo group (*d* = 1.907). For the group with a medium connection, we report a significant increase of connection to nature for the guided tour without addition, the tour with a keeper talk, the tour with a view behind the scenes, and the tour with feeding giraffes. While the first three showed a moderate effect size *d* < 1.0, the effect of feeding giraffes was notably higher (*d* = 1.310) (Figure 4 and Table 3).

**TABLE 3:** Results of the Wilcoxon tests with mean scores for T1 and T2 and effect (*d*) size for the different experimental groups.

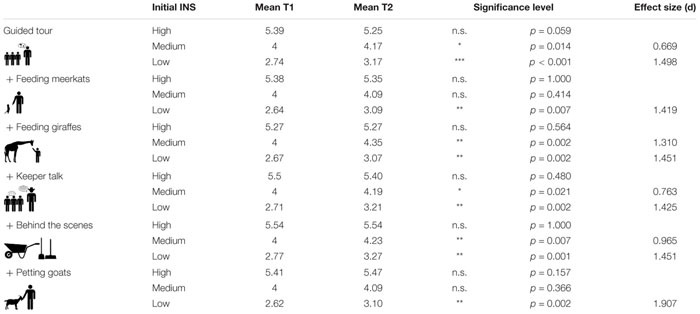

Significant shifts are marked: *p < 0.05,**p < 0.01, ***p < 0.001.

### Discussion

Our results clearly show that students with an initially lower INS score – regardless of the type of environmental education program – have a positive influence on their proximity to nature. Even the most basic environmental education program in a zoo, a 1 h guided tour, has the potential to increase the connection to nature with a large effect size. There are two main ways to connected people with nature that are discussed in environmental literature: on the one hand, the time spent in nature is an important predictor of the connection to nature. Several studies show that people who spend more time in nature have a higher connection to nature (Schultz and Tabanico, 2007; Mayer et al., 2009; Nisbet et al., 2009). Furthermore, experiences in nature influence connection to nature (Kals et al., 1999). Even though zoo enclosures are artificially designed environments, they contain many naturalistic elements visitors can see and experience. Observing wild animals in naturalistic exhibits contributes to immerse into the habitat and the visitors a special experience that has the ability to increase connection to nature. On the other hand, it is frequently reported that environmental education is a possibility to connected people with nature. For example, Braun and Dierkes (2017) demonstrated a positive effect of an environmental education program on student’s connection to nature. For younger students, the effect was more stable than for older ones. Similar results were reported by Liefländer et al. (2013). Kossack and Bogner (2012) explained the positive shift of connection to nature by their environmental education program with the participants’ gain of knowledge. Besides a lot of interesting information about the different animal species, the guided zoo tour also included knowledge that is particularly relevant for biology education, making the tour an adequate environmental education program for upper secondary school students.

Other factors could also be responsible for the large increase in the group with initial low connection to nature. For example, there is a positive correlation between previous experiences of nature, motivation to participate in nature-based activities, and connection with nature (Cheng and Monroe, 2012). It can therefore be assumed that students with less connection to nature also have less direct contact with nature. The direct contact with nature, as it took place at the zoo, is therefore potentially a new experience for this group, which may have led to a strong change in their relationship with nature. For nature-connected, on the other hand, contact with nature was not a new experience, so there was no significant change.

In addition, social interactions in school classes could have an influence on the increase in connection with nature, especially among students with a low level of connection with nature. A number of theories describe the special role and influence of peers on a person’s behavior and attitude (Gross-Manos, 2014). It is therefore quite conceivable that the positive attitude of the students with a high degree of connection to nature was transferred to the students with a low degree of connection to nature and thus contributed to the increase.

Students with an initial medium connection to nature show the clearest distinction between the different test settings. Although the guided tour without addition led to a significant increase, no significant effects were observed in the guided tour with feeding meerkats and the guided tour with petting goats. From the perspective of the connection to nature, the add-ons seem to have counteracted the positive effect of the guided tour on this group. There are several explanations for this result. When feeding the meerkats, it is possible that the mealworms disgusted the students. A negative experience could negatively influence connection to nature. Furthermore, the participants were not allowed to touch the meerkats because as a predator it was likely that they would try to bite the students. Perhaps the combination of these circumstances disappointed the students and prevented this group from feeling more connected to nature. For the group that petted goats, the explanation is different. The basic idea of this add-on was that direct contact with the animals would strengthen the connection to nature. While some students were excited to pet the goats, others were less interested and felt too old for the petting zoo. Furthermore, the petting zoo is open to all visitors, so that a visit to the petting zoo, unlike the other add-ons, was not a special experience.

However, for the students with initial low connection to nature, petting goats showed an additional increase of connection to nature compared to the guided tour without an add-on. This result could be explained by the high affective component in feeding the goats. Kossack and Bogner (2012) suggest that students with an initially low connection to nature should be encouraged mainly by emotional elements. Feeding and petting of goats, including juvenile animals, may have addressed the emotional component in these participants, which would explain an additional increase in connection to nature compared to the control group.

The largest effect size for students with an initially medium connection to nature was obtained in the tour with feeding giraffes (*d* = 1.310) and is probably due to the high attractiveness of these animals and the direct contact during feeding. From 40 different species tested at Chester Zoo, most visitors stopped at the giraffe enclosure and spend most of their time observing the giraffes (Moss and Esson, 2010). Body size and attractiveness to visitors are, among other factors, important predictors for zoos that play a role in the selection of species for their animal collection (Frynta et al., 2013). Visitors feel particularly attached to large zoo animals (both herbivores and carnivores). This connection with the animals often correlates with the concern for the protection of these animals (Howell et al., 2019). An extraordinary experience with these rare and special animals seems to have a positive effect on connection to nature, especially for participants with an initial medium connection.

Participants with an initially high connection were almost unaffected in the different test settings. However, this effect is not unusual and is also reported in other studies (Braun and Dierkes, 2017). It is obviously difficult to achieve an additional positive effect in people with a high level of connection to nature, as they already have a high level of initial connection to nature. Although it is desirable to further increase the connection to nature of students with an initial high connection to nature, the focus of environmental education programs should, however, be on students who are less close to nature, because an environmentally friendly behavior increases with connection to nature (Dunlap et al., 2000; Clayton, 2003; Nisbet et al., 2009) and a split between humans and nature leads to environmental problems (Jordan, 2009).

## Limitation and Further Research

Our study has a very strict focus on connection to nature. Even when we report no significant increase of connection to nature for some sample groups, it is possible that other unobserved variables changed during the guided tour. As described in the introduction, environmental behavior, environmental knowledge, or environmental attitudes also play an important role, and it is possible that certain interventions have a particular influence here. For example, the keeper talk might have led to additional knowledge acquisition, or petting the goats might have increased their connection to animals. A change in attitude toward zoos, more precisely a more positive view, or the establishment of a more differentiated approach to animal husbandry in zoos (for instance by the keeper interview) is also possible, but was not recorded in our study. Further research is required to investigate effects on these unobserved but important variables.

Another factor that was not considered in our study is the number of visits to the zoo before the intervention. So it is quite conceivable that there is a connection between the regularity of zoo visits and the closeness to nature. For example, students with a lower level of connection to nature may visit the zoo less often in their free time than students with a high level of connection to nature. Further research in this context would be useful to explore the longer-term role of zoos in strengthening human–nature relationships. The observation of the teacher could also be a starting point for the following studies. It is possible, for example, that a teacher who is highly motivated and integrates the visit to the zoo specifically into the lessons could have a positive influence on the results of the study.

Many studies show that the connection to nature depends strongly on age. Older students have lower INS scores than younger students (Schultz and Tabanico, 2007; Fröhlich et al., 2013; Liefländer and Bogner, 2014; Sattler, 2016; Braun and Dierkes, 2017). Hughes et al. (2019) analyzed this relationship between age and connection to nature in a cross-sectional study with more than 2,300 participants. They found that children younger than 12 years have a particularly high level of connection to nature, but this decreases with increasing age until it reaches the lowest value for young adults. Our study focused on this age group, where, apart from the low connection to nature, the interest in animals and especially in zoo-specific topics is lower. It is quite possible that the tour or the respective add-on will show different results or even stronger effects for other age groups. Interestingly, our programs did not show significant increases in students with an initially high connection to nature. In this context, it would be interesting to evaluate whether the temporal component has a decisive influence. The guided tour with a maximum duration of 1.5 h is a short-term intervention. It has already been shown that longer-term environmental education programs can also lead to an increased connection to nature among these pupils (Braun and Dierkes, 2017).

## Conclusion

The study showed that the interest in plants and animals, and especially zoo animals, strongly decreases during the education period. Especially high school students show a particularly low interest in these topics compared to other areas such as health or environmental protection. This is consistent with the results of previous studies that found a decline in the human–nature relationship for this age group (Hughes et al., 2019). It is therefore particularly rewarding to offer environmental education programs with a focus on animals and plants for this age group of students.

The results show that a 1 h guided tour at a zoo increases connection to nature for an age group that is less interested in the topic of zoo and animals. Especially students with an initially low or medium level of connection to nature benefit from the guided tours. For this reason, guided tours seem to be an efficient and meaningful zoo pedagogical instrument in the field of environmental education, even though they require a lot of time and staff. Small additions to the tour can improve the result, but not all add-ons show similar positive effect.

## Data Availability Statement

The raw data supporting the conclusions of this article will be made available by the authors, without undue reservation.

## Ethics Statement

Ethical review and approval was not required for the study on human participants in accordance with the local legislation and institutional requirements. Written informed consent to participate in this study was provided by the participants’ legal guardian/next of kin.

## Author Contributions

PD, MK, NH, and MB: conceptualization. JG, AP, CD, and TS: data collection. PD, NH, and MK: methodology. MK: validation, formal analysis, investigation, and writing – original draft preparation. MK and PD: writing – review and editing and visualization. PD: funding acquisition. All authors contributed to the article and approved the submitted version.

## Conflict of Interest

The authors declare that the research was conducted in the absence of any commercial or financial relationships that could be construed as a potential conflict of interest.

## Figures and Tables

**Appendix 1:**
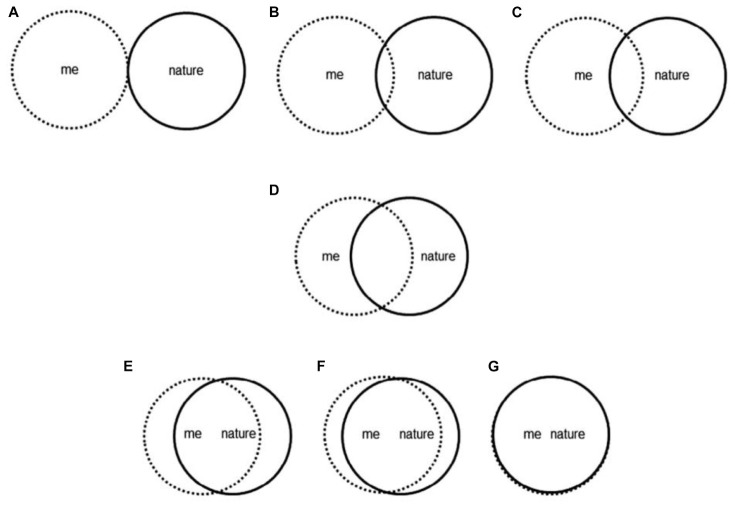
INS.

**Appendix 2:** Mean values ± standard deviation of the different grades according to items.

			**5**	**6**	**7**	**9**	**10/11**	**12**	**13**
Animals	A1	What zoos do for protection of species	2.91 ± 1.06 (408)	2.66 ± 1.07 (61)	2.30 ± 1.04 (412)	2.01 ± 0.92 (438)	1.73 ± 0.91 (41)	1.91 ± 0.94 (174)	1.76 ± 0.78 (33)
	A2	Animals in other areas of the world	3.23 ± 0.93 (407)	3.16 ± 1.04 (61)	2.79 ± 1.00 (410)	2.49 ± 0.97 (443)	2.24 ± 0.85 (41)	2.38 ± 0.93 (173)	2.36 ± 1.01 (33)
	A3	Animals in the zoo	2.85 ± 1.10 (409)	2.45 ± 1.09 (62)	2.32 ± 1.07 (417)	1.89 ± 0.97 (446)	1.71 ± 0.89 (41)	1.94 ± 1.01 (171)	1.75 ± 0.83 (32)
	A4	Behaviors of animals	3.17 ± 0.95 (408)	2.70 ± 1.09 (61)	2.74 ± 1.08 (413)	2.59 ± 0.94 (442)	2.46 ± 0.99 (41)	2.45 ± 0.96 (174)	2.76 ± 0.82 (33)
	A5	How to protect endangered species	3.38 ± 0.78 (410)	3.10 ± 0.91 (60)	2.99 ± 0.86 (417)	2.84 ± 0.86 (444)	2.78 ± 0.88 (40)	2.79 ± 0.85 (174)	3.00 ± 0.74 (33)
Plants	P1	Plants in my environment	2.52 ± 1.01 (410)	1.95 ± 1.09 (60)	1.93 ± 0.89 (411)	1.83 ± 0.80 (443)	1.76 ± 0.69 (41)	1.71 ± 0.79 (174)	1.67 ± 0.68 (33)
	P2	How plants grow and multiply	2.52 ± 1.02 (407)	1.98 ± 1.08 (62)	1.96 ± 0.87 (416)	1.94 ± 0.81 (444)	1.83 ± 0.76 (41)	1.79 ± 0.74 (174)	1.76 ± 0.70 (33)
	P3	Plants and their benefits for humans	2.75 ± 1.04 (409)	2.22 ± 1.05 (60)	2.21 ± 0.94 (413)	2.28 ± 0.88 (442)	2.13 ± 0.93 (40)	2.20 ± 0.82 (171)	2.39 ± 0.92 (33)
	P4	How plants produce food	2.88 ± 0.96 (404)	2.51 ± 1.00 (61)	2.44 ± 0.92 (441)	2.37 ± 0.88 (445)	2.22 ± 0.78 (41)	2.37 ± 0.90 (172)	2.34 ± 1.02 (32)
Health	H1	What you have to eat to stay healthy	3.21 ± 0.98 (407)	2.90 ± 0.85 (60)	3.16 ± 0.97 (414)	3.28 ± 0.92 (442)	3.39 ± 0.73 (41)	3.24 ± 0.93 (174)	3.33 ± 0.80 (33)
	H2	How to train to keep the body fit and healthy	3.29 ± 0.95 (408)	2.87 ± 1.05 (62)	3.39 ± 0.83 (412)	3.44 ± 0.81 (443)	3.48 ± 0.87 (40)	3.43 ± 0.87 (174)	3.42 ± 0.78 (33)
	H3	Nutrition of humans	2.61 ± 1.03 (409)	2.48 ± 1.07 (61)	2.46 ± 0.96 (416)	2.72 ± 0.98 (443)	3.02 ± 0.90 (41)	2.98 ± 1.00 (174)	2.91 ± 1.03 (33)
Environment	E1	The greenhouse effect and how humans can change the situation	2.59 ± 1.09 (352)	2.34 ± 1.15 (47)	2.42 ± 1.02 (358)	2.62 ± 0.95 (416)	2.84 ± 1.01 (38)	2.89 ± 1.01 (174)	2.79 ± 1.07 (33)
	E2	How to preserve and protect biodiversity in the world	2.88 ± 1.04 (404)	2.57 ± 1.10 (56)	2.42 ± 0.97 (405)	2.44 ± 0.98 (443)	2.48 ± 0.92 (40)	2.56 ± 0.97 (174)	2.76 ± 0.95 (33)
	E3	Habitat earth	3.02 ± 0.96 (409)	2.83 ± 1.07 (60)	2.69 ± 0.94 (414)	2.72 ± 0.84 (442)	2.78 ± 0.81 (41)	2.55 ± 0.81 (173)	2.52 ± 0.86 (33)

**The number of participants is indicated in brackets.**
